# Identification of RAC1 in promoting brain metastasis of lung adenocarcinoma using single-cell transcriptome sequencing

**DOI:** 10.1038/s41419-023-05823-y

**Published:** 2023-05-18

**Authors:** Mingyu Chen, Hanyue Li, Xiaolin Xu, Xunxia Bao, Lei Xue, Xinghao Ai, Jian Xu, Ming Xu, Yong Shi, Timing Zhen, Jie Li, Yi Yang, Yang Ji, Zhiliang Fu, Kaichen Xing, Tao Qing, Qiubo Wang, Ping Zhong, Sibo Zhu

**Affiliations:** 1 Department of Neurosurgery, Huashan Hospital, Shanghai Medical College, Fudan University, 200040 Shanghai, China; 2 School of Life Sciences, Fudan University, 200438 Shanghai, China; 3 Research Unit of New Technologies of Micro-Endoscopy Combination in Skull Base Surgery (2018RU008), Chinese Academy of Medical Sciences, Beijing, China; 4 Neurosurgical Institute of Fudan University, Shanghai, China; 5 Shanghai Clinical Medical Center of Neurosurgery, Shanghai, China; 6 Shanghai Key Laboratory of Brain Function Restoration and Neural Regeneration, Shanghai, China; 7 Department of Lung Tumor Clinical Center, Shanghai Chest Hospital, Shanghai Jiaotong University, 200030 Shanghai, China; 8 Department of Cardiothoracic Surgery, Third Affiliated Hospital of Naval Military Medical University, 200003 Shanghai, China; 9 Department of Thoracic Surgery, Shanghai Pulmonary Hospital, Tongji University, 507 Zhengmin Road, Shanghai, PR China; 10 School of Life Science, Anhui Medical University, 230032 Hefei, China; 11 Department of Thoracic Surgery, Shanghai Changzheng Hospital, Second Affiliated Hospital of Naval Military Medical University, 200003 Shanghai, China; 12 Cinoasia Institute, 200438 Shanghai, China; 13 Department of Clinical Laboratory, Wuxi 9th People’s Hospital Affiliated to Soochow University, 214000 Wuxi, Jiangsu China

**Keywords:** Non-small-cell lung cancer, Mechanisms of disease, Predictive markers, Metastasis

## Abstract

This study aims to give a new perspective to the biomarkers in the lung adenocarcinoma (LUAD) brain metastasis, pathways involved and potential therapeutics. We performed a comprehensive single-cell level transcriptomic analysis on one LUAD patient with circulating tumor cells (CTCs), primary tumor tissue and metastatic tumor tissue using scRNA-seq approach to identify metastasis related biomarkers. Further scRNA-seq were performed on 7 patients to validate the cancer metastatic hallmark. with single cells collected from either metastatic or primary LUAD tissues. Pathological and functional studies were also performed to evidence the critical role of RAC1 in the LUAD metastasis. Hallmark gene was verified based on immunohistochemistry staining, cytological experiment, survival information from The Cancer Genome Atlas (TCGA), and staining results from Human Protein Atlas (HPA) databases. PCA analysis revealed that CTCs were in the intermediate place between the metastatic group and primary group. In the unsupervised clustering analysis CTCs were closer to one of the metastatic tumor cells, implying heterogeneity of the metastatic tumor and origin of the CTCs were from metastatic site. Transitional phase related gene analysis identified RAC1 was enriched in metastatic tumor tissue (MTT) preferred gene set functioning as regulated cell death and apoptosis as well as promoted macromolecule organization. Compared with normal tissue, expression levels of RAC1 increased significantly in LUAD tissue based on HPA database. High expression of RAC1 predicts worse prognosis and higher-risk. EMT analysis identified the propensity of mesenchymal state in primary cells while epithelial signals were higher in the metastatic site. Functional clustering and pathway analyses suggested genes in RAC1 highly expressed cells played critical roles in adhesion, ECM and VEGF signaling pathways. Inhibition of RAC1 attenuates the proliferation, invasiveness and migration ability of lung cancer cells. Besides, through MRI T2WI results, we proved that RAC1 can promote brain metastasis in the RAC1-overexpressed H1975 cell burden nude mouse model. RAC1 and its mechanisms might promote drug design against LUAD brain metastasis.

## Introduction

Lung adenocarcinoma (LUAD) is the most prevalent non-small cell lung cancer (NSCLC) cancer type that accounts for 85% of the lung cancer with a very low 5-year survival rate. The brain is the main organ prone to LUAD metastasis, and there is no effective treatment against metastasis due to unknown mechanisms [1, 2].

Genomic analysis revealed a series of significant exon mutation load in the brain metastases such as PI3K/AKT/mTOR, CDK, and HER2/EGFR which are likely to induce primary lung cancer develop into metastases. More recently, transcriptomic data revealed that WNT/TCF pathway play an important role in the metastasis [3, 4]. TGFβ is dysregulated in malignant cells of lung cancer, and TGFβ up-regulation in the ECM of lung cancer promotes tumor progression and invasion [5]. Downregulation of Alkaline phosphatase decreased ERK phosphorylation in LUAD, causing an accumulation of c-Myc, which promotes RhoA transcription, leading to increased metastasis of LUAD cells [6]. However data from these metastasis studies were based on cell biology, immunochemistry or bulk omics approach which was likely to mask features from subpopulations [7].

Single-cell RNA-seq (scRNA-seq) is a technology that deciphers cells isolated from bulk into individual cells as well as CTCs, to reliably distinguish neoplastic from non-neoplastic cells, to correlate signaling pathways between neoplastic cells and stroma, and to map expression signatures to inferred clones and phylogenies [8]. CTCs are extraordinarily rare, metastasizing cells in the circulation, with 1–10 cells per ml blood [9, 10]. After dissociated from solid tumor, CTCs travel in the vasculature as single cells or clusters to form distant metastases, probably based on the Epithelial-mesenchymal transition (EMT) procedure [11]. However what are the molecule patterns in the CTCs compared to primary and metastatic cancerous cells and whether the hallmarks from metastatic cells share common traits across patients with metastasis remain unknown by far.

To understand the mechanisms and molecules promoting metastasis of LUAD, we performed a single-cell level transcriptomic analysis on a LUAD patient with primary cancer biopsy, CTC and metastatic cancer tissue using SMART-Seq2 scRNA-seq approach [12]. We found that RAC1 was highly enriched in metastatic cells, and further expand datasets to 8 patients with single cells collected from either metastatic or primary LUAD tissues. Pathological and functional studies were also performed to evidence the critical role of RAC1 in the LUAD metastasis. This study aims to give a new perspective to the biomarkers in the LUAD brain metastasis, pathways involved and potential therapeutics.

## Results

### Single patient’s sample data analysis showed that RAC1 was highly enriched in the metastatic cells

**Fig. 1:**
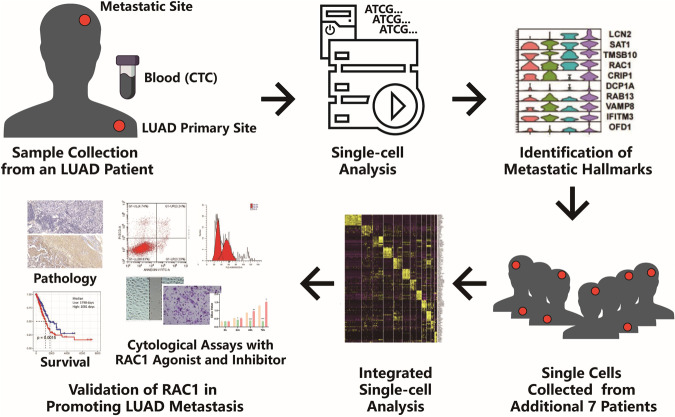
Overview of the study design. A total 168 single cells from primary tumor tissue (PTT), primary para-tumor tissue (PTP), metastatic tumor tissue (MTT), para-metastatic tumor normal brain tissue (MTP), and circulating tumor cell (CTCs) from a LUAD patient was collected and sequenced by smart-seq2 RNA sequencing method. The data analysis was performed to find the hallmarks of metastasis related genes including RAC1. Further 7 patients with either primary tumors or metastatic tumors were collected for expansion of the single-cell data set. Pathological sections of additional six patients were further applied for RAC1 IHC staining. TCGA and survival data were also employed to find the relationship between the function of RAC1 and LUAD.

We first sequenced 168 single cells from an LUAD patient (Patient1) with primary tissue, CTC and metastatic tissue. After filtration and trimming with criteria in the pipeline, 124 out of 168 samples passed quality control. A general overview of the study design and sample information are shown in Fig. 1, Fig. S1, and Table 1.

**Table 1:** Samples from nine LUAD patients.

Tissue	PT1	PT2	PT3	PT4	PT6	PT7	PT8	PT9	Sum
CTC	16	0	0	0	0	0	0	0	16
MTP	30	0	0	24	16	29	0	20	119
MTT	43	0	0	63	68	31	28	39	272
PTP	13	0	0	19	0	0	0	0	32
PTT	22	47	55	56	0	0	0	0	180
sum	124	47	55	162	84	60	28	59	619

**Fig. 2:**
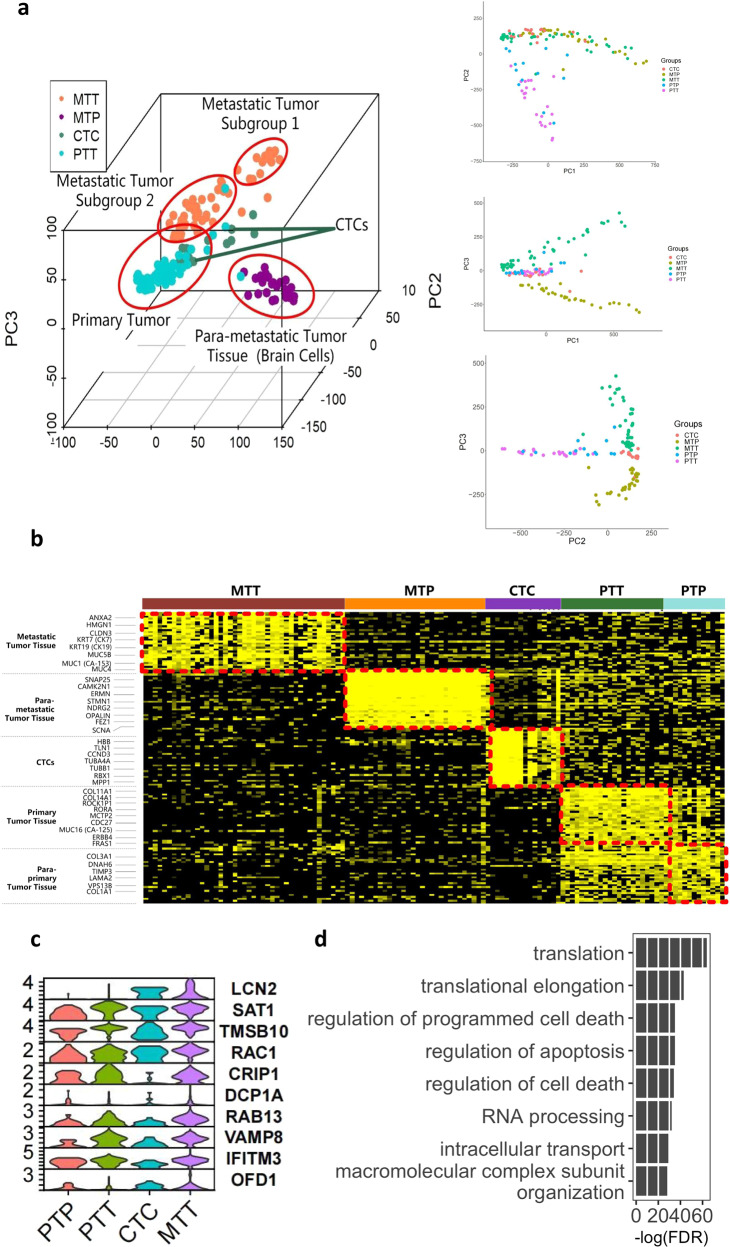
Single cell analysis of 1 LUAD patient. **a** PCA unveiled several subgroups of single cells as was determined by tissue of origins including primary tumor cells, primary para-tumor cells, metastatic tumor subgroup 1, metastatic tumor subgroup 2, para-metastasis brain tissue, and CTC. **b** Heatmap of tissue-specific markers was plotted and previous reported features were identified in the data set. **c** Genes with elevating expression levels from primary to CTC to metastatic sites. **d** Functional annotation indicated MTT favored genes functions as regulated cell death and apoptosis as well as promoted macromolecule organization.

PCA was first plotted to present distribution of the single cells from primary tumor, metastatic tumor, para-metastasis brain tissue, and CTCs (Fig. 2a). Interestingly, CTCs were closer to metastatic tumor in PC1/2 yet closer to primary tumor in PC1/3. CTCs were also shown in between the metastatic group and primary group in PC2/3, implying the transitional or intermediate state of the cancer during metastasis. Despite the heterogeneity across the cells, most of the samples were clustered as their tissue of origins which is likely due to small sample size. We then analyzed tissue specific markers based on differentially expressed genes and heatmap was plotted (Fig. 2b).

We hypothesized that CTC functioned as a transitional state during primary-to-metastatic phase and metastasis promoting pathways were likely to be enhanced in the pathogenesis. We analyzed significantly enhanced metastasis-to-CTC and CTC-to-primary DEGs to find a group of continuously upregulated genes (Table S1). Genes such as LCN2, SAT1, RAC1, IFITM3, VAMP8, RAB13, NFKBIA and S100A4 were elevated in CTCs and even higher in the MTT cells. Violin plots represented the expression profile of gradually up-regulated genes in “primary tumor tissue (PTT)-CTC-MTT” (Fig. 2c). Functional annotation indicated these metastatic related geneset played critical roles in regulated cell death and apoptosis as well as promoted macromolecule organization (Fig. 2d). According to previous literature, RAC1 is inseparably important for cell motility and contributes to crawling, invasion, proliferation, anti-apoptosis, etc.

In order to reveal EMT states, we used EMT signatures to quantify the Epithelial and Mesenchymal score in each cancer single cell [13–15] (Fig. S2a). Heatmap of EMT signatures showed distinctive patterns in three cancer populations. Proportion of Epithelial signal was higher in MTT, while PTT showed a propensity of Mesenchymal state (Fig. S2b, c). CTCs expressed either low E+ or M+ scores, probably due to transitional state in the blood. We further expanded the dataset and investigated Epithelial markers, Mesenchymal markers and EMT related pathways hallmarks using 452 cells (272 cells from metastatic cancer cells and 180 from primary cancer cells) of all 8 patients. Results revealed a higher Mesenchymal markers in primary cancer cells (e.g., NCAM1, LAMB1, SERPINE1, TCF4, VIM, ZEB1, and ZEB2) and a higher Epithelial markers in metastatic cancer cells (e.g., CDH1, CLDN4, ELF3, EPCAM, KRT18, KRT7, and KRT8). It is interesting a few EMT Pathway signaling molecules were upregulated in metastatic cells including AKT1, MAPK1, MAPK3, RAS genes (NRAS, KRAS, and HRAS), ROS1, and PTK2 were up-regulated (Fig. S3).

### Combined analysis of multiple patient samples to explore RAC1-related genes and pathways

**Fig. 3:**
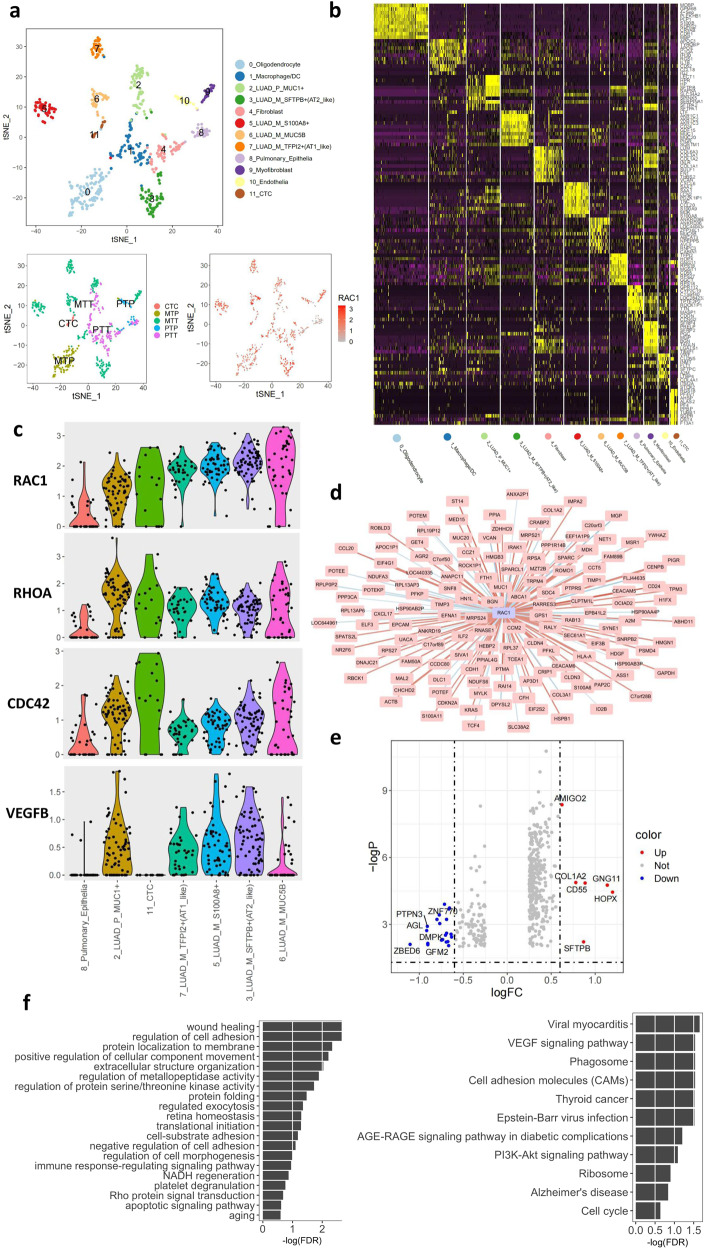
Single cell analysis of 8 LUAD patients. **a** T-SNE plot of single cells from 8 patients. Additional 7 patients were enrolled for single cell sequencing by using either primary or metastatic tissues. The cells were clustered using T-SNE to obtain 12 stable subgroups, among with RAC1 was highly expressed in the metastatic cancer cells. **b** Each subgroup showed its unique signature genes. **c** High expression of RAC1, RHOA, CDC42, and VEGFB were identified in metastatic subgroups, compared to normal clusters. **d** Co-expression network showed genes closely associated with RAC1. **e** The volcano map showed deferentially expressed genes in tumor cells between RAC1^high^ and RAC1^low^ expressing cells. **f** Functional clustering and pathway analyses suggested genes in RAC1 highly expressed cells played critical roles in adhesion, ECM and VEGF signaling pathways.

In order to validate the metastasis related molecules, we recruited additional 7 patients were single-cell sequencing by using either primary or metastatic tissues. The T-SNE plot of single cells from 8 patients showed that cells were clustered into 12 stable subgroups, among with RAC1 was highly expressed in the metastatic cancer cells (Fig. 3a). Heatmap was plotted and each subgroup showed its unique signature genes (Fig. 3b). High expression of RAC1, and RAC1 related genes including RHOA, CDC42, and VEGFB were identified in metastatic subgroups and CTCs, compared to primary tumor and normal lung epithelia clusters (Fig. 3c). Co-expression network showed genes closely associated with RAC1 were calculated among which cancer genes such as MUC1, MUC20, CEACAM5, andCCL20 were positively correlated to expression of RAC1 whereas TIMP3 and MGP was negatively correlated (Fig. 3d and Fig. S4). The volcano map showed deferentially expressed genes in tumor cells between RAC1^high^ and RAC1^low^ expressing cells (Fig. 3e). Functional clustering and pathway analyses suggested genes in RAC1 highly expressed cells played critical roles in adhesion, ECM and VEGF signaling pathways (Fig. 3f).

To demonstrate the evolution of LUAD, we plotted a graph based on Monocle 2. The solid black line represented the main route of the minimal spanning tree constructed, which exhibited the backbone and order of the LUAD development along a pseudo-temporal continuum. The trajectory clearly showed the tree starts from 8_Pulmonary_Epithelis, goes through subgroups 6, 11, 3, 2, 7 and ends with 5_LUAD_M_S100A8. Based on the pseudo-temporal continuum profile, we identified representative GOBP and KEGG concentrating terms based on regulated kernel evolutionary enriched genes functional annotations (Fig. S5).

### RAC1 is the marker of metastasis and represents a poor prognosis

**Fig. 4:**
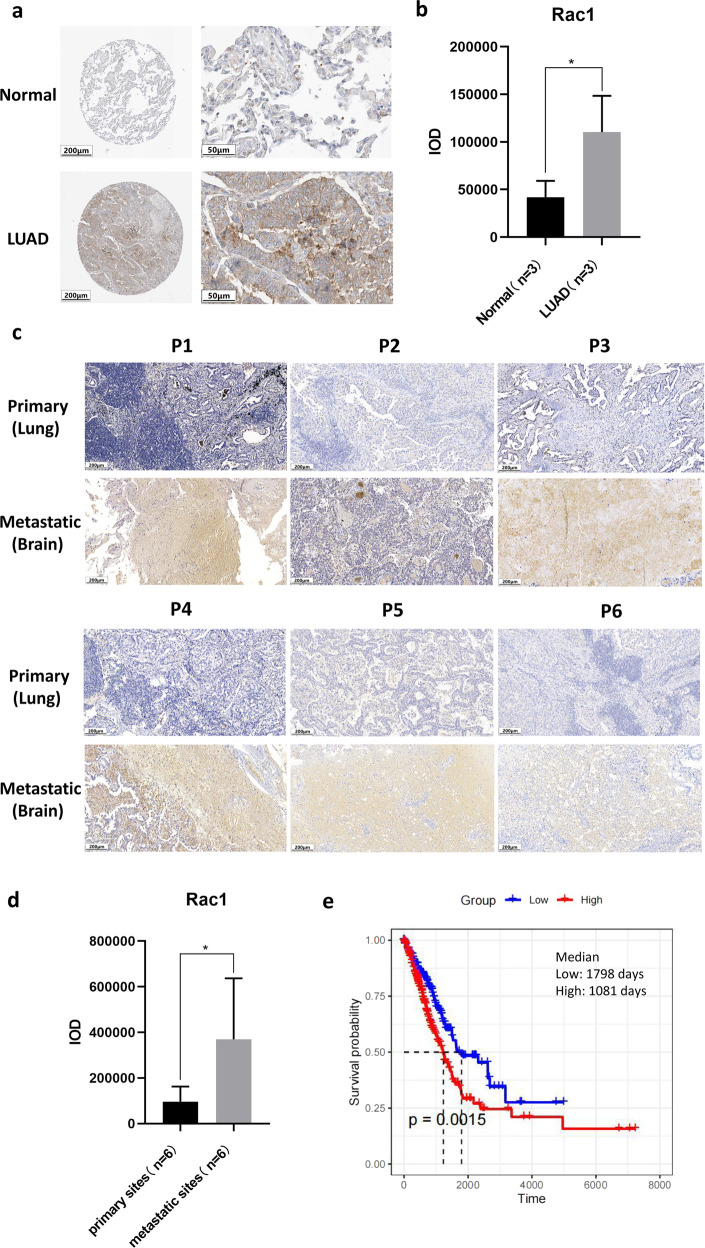
Pathological evidence of LUAD patients. **a**, **b** Compared with normal tissue, expression level of RAC1 (IOD, Integrated Optical Density) increased significantly in LUAD tissues, based on HPA database (normal tissue, *n* = 3; LUAD, *n* = 3; Fold change = 2.63; *P* < 0.01). **c**, **d** Additional tissue samples from 6 patients with both primary and metastatic LUAD were used for RAC1 staining analysis. IOD results showed high expression of RAC1 in the metastases (normal tissue, *n* = 6; LUAD, *n* = 6; Fold change = 3.83; *P* < 0.05). **e** Survival analyses of RAC1 expression in LUAD patients. The red line represents patients with higher RAC1 and the blue line denotes patients with lower RAC1 expression. The survival time of LUAD patients with high RAC1 expression was lower (red line) with median survival of 1081 days, compared to a more favorable survival of patients with low RAC1 expression (1798 days, *P* = 0.0015).

The pathological evidence of RAC1 were also obtained from public RNA and protein datasets and IHC staining from our LUAD patients. We first analyzed images of LUAD patients from HPA datasets. Compared with normal tissue, the Integrated Optical Density (IOD) level of RAC1 increased significantly in LUAD tissues (normal tissue, *n* = 3; LUAD, *n* = 3; Fold change = 2.63; *P* < 0.01) (Fig. 4a, b). Additional tissue samples from 6 patients with both primary and metastatic LUAD were used for RAC1 staining analysis. IOD results showed high expression of RAC1 in the metastases (normal tissue, *n* = 6; LUAD, *n* = 6; Fold change = 3.83; *P* < 0.05) (Fig. 4c, d). By using TCGA datasets (Oncolnc online tool), we analyzed survival based on the expression of RAC1 in LUAD patients. The survival time of LUAD patients with high RAC1 expression was lower (red line) with median survival of 1081 days, compared to a more favorable survival of patients with low RAC1 expression for 1798 days (*P* = 0.0015) (Fig. 4e).

### Expression levels of RAC1 accelerate the growth and metastasis of lung cancer cells from multiple perspectives

**Fig. 5:**
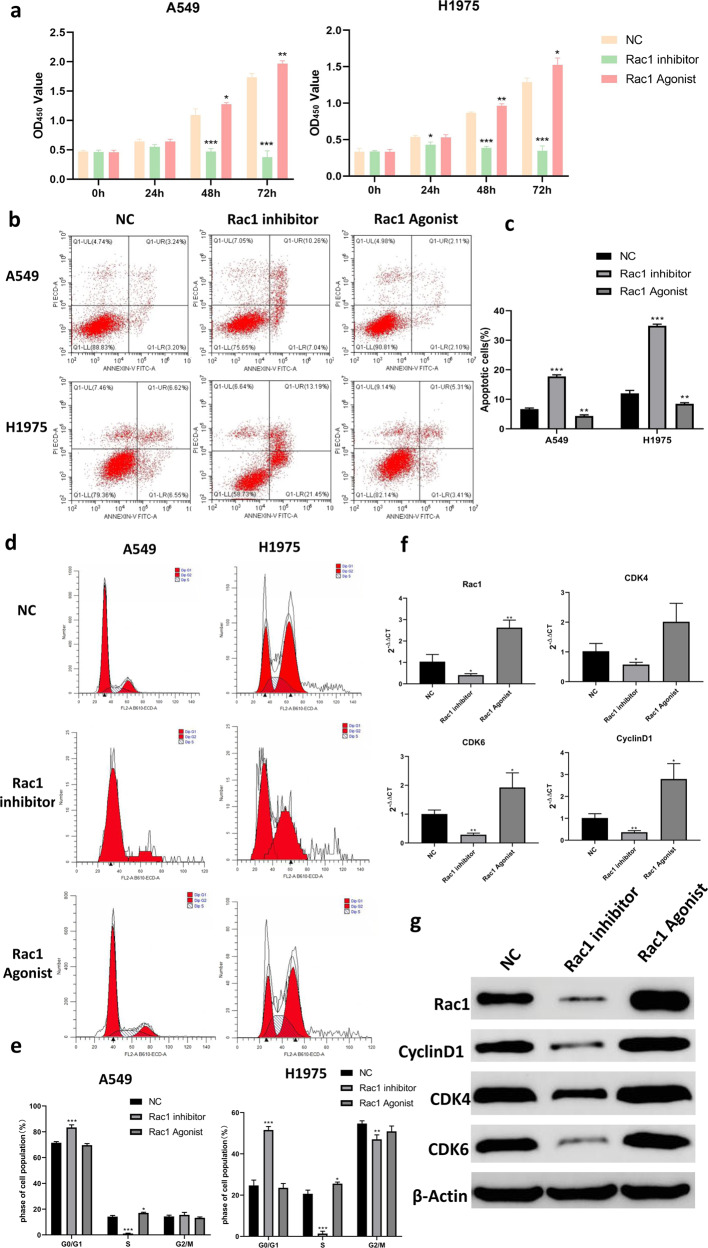
RAC1 promotes the proliferation and cell cycle of tumor cells. **a** CCK-8 assay showed that RAC1 inhibitor could significantly slowed down the proliferation of A549 (Left panel) and H1975 (Right panel) cells at 48 h and 72 h (*P* < 0.01) while RAC1 agonist slightly promoted the growth of cell lines. **b**, **c** Apoptosis analyses by using AnnexinV-PI and flow cytometry assay showed RAC1 inhibitor significantly promoted apoptosis of both LUAD cell lines (*P* < 0.001 for H1975; *P* < 0.001 for A549). Proportion of apoptotic cells were reduced by RAC1 agonist in the cell lines (*P* < 0.01 for H1975; *P* < 0.01 for A549). **d**, **e** RAC1 inhibitor promoted apoptosis of the two LUAD cells and impeded the transition from G0/G1 phase to S phase (*P* < 0.001 for enhanced G0/G1 phase, *P* < 0.001 for suppresed S phase), thereby preventing cell division. **f**, **g** The qPCR and WB results also showed that RAC1 inhibitors could reduce the expression of cycle-related genes and proteins (CDK4,CDK6, CyclinD1) and RAC1 agonists could promote the expression of cycle-related genes, which is consistent with the findings of previous related studies. Data are expressed as means ± SD; *P < 0.05, **P < 0.01, ***P < 0.001.

**Fig. 6:**
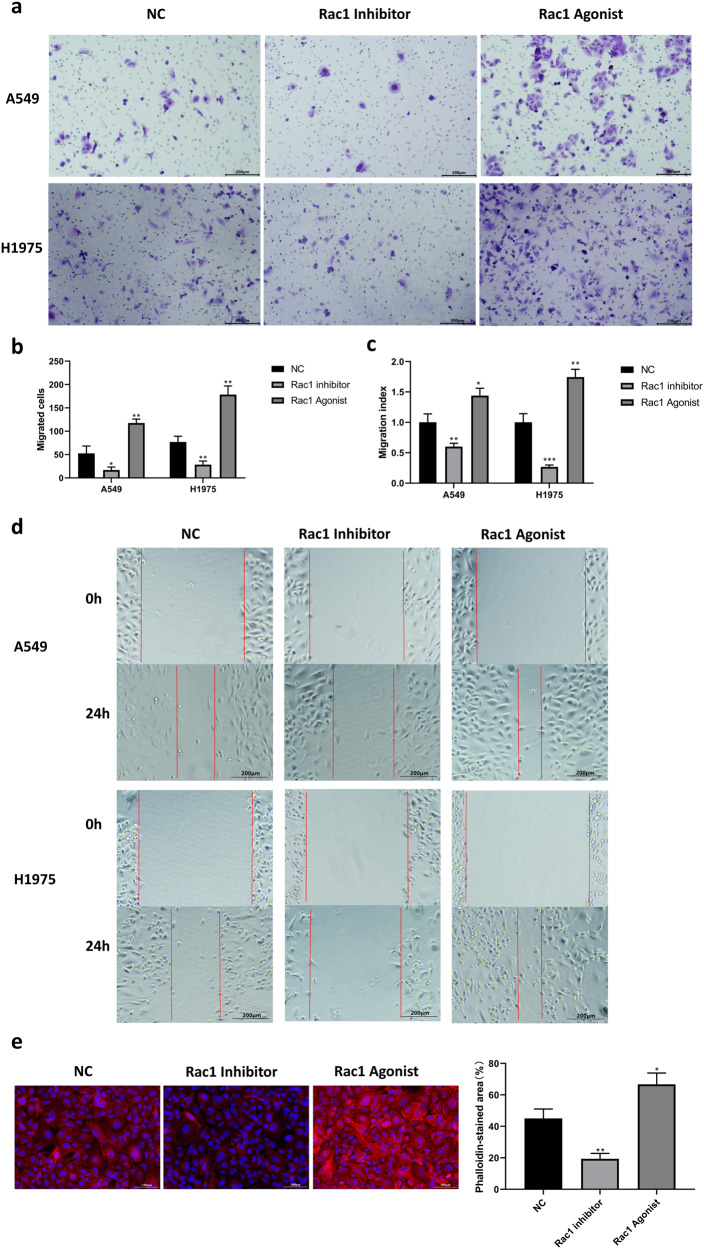
RAC1 promotes the invasiveness and migration ability of LUAD cells. **a**, **b** Activated RAC1 function is validated to significantly improve the ability of cell invasion (*P* < 0.01 for H1975; *P* < 0.05 for A549) by Transwell assay, while cells with disrupted RAC1 function were present to be inactive (*P* < 0.05 for H1975; *P* < 0.05 for A549). **c**, **d** Scratch wound healing test showed that activated RAC1 could enhance the migration ability of lung cancer cells in the plate (*P* < 0.01 for H1975; *P* < 0.05 for A549). However A549 (*P* < 0.01) and H1975 (*P* < 0.001) recovers wound more slowly in the presence of RAC1 inhibitor. **e** The phalloidin staining results showed that RAC1 inhibitor could significantly inhibit the expression of polymeric actin in lung cancer (*P* < 0.01), and RAC1 agonist agent could significantly promote the expression of polymeric actin in lung cancer cells (*P* < 0.05). Data are expressed as means ± SD; *P < 0.05, **P < 0.01, ***P < 0.001.

We adopted LUAD cell lines to further identify the invasiveness by using cytological assays. CCK-8 assay showed that RAC1 inhibitor could significantly slowed down the proliferation of A549 and H1975 cells at 48 h and 72 h (*p* < 0.01) while RAC1 agonist slightly promoted the growth of cell lines (Fig. 5a). Apoptosis (Fig. 5b, c) analyses by using AnnexinV-PI and flow cytometry assay showed RAC1 inhibitor significantly promoted apoptosis of both LUAD cell lines (*P* < 0.001 for H1975; *P* < 0.001 for A549). Proportion of apoptotic cells were reduced by RAC1 agonist in the cell lines (*P* < 0.01 for H1975; *P* < 0.01 for A549). In terms of the cell cycles, RAC1 inhibitor impeded the transition from G0/G1 phase to S phase (*P* < 0.001 for enhanced G0/G1 phase, *P* < 0.001 for suppressed S phase), thereby preventing cell division (Fig. 5d, e). The qPCR and WB results also showed that RAC1 inhibitors could reduce the expression of cycle-related genes and proteins (CDK4,CDK6, CyclinD1) while RAC1 agonists could promote the expression of cycle-related genes (Fig. 5f, g). Activated RAC1 function is validated to significantly improve the ability of the two cell lines’ invasiveness (*P* < 0.01 for H1975; *P* < 0.05 for A549) by Transwell assay (Fig. 6a, b), while cells with disrupted RAC1 function were present to be inactive (*P* < 0.05 for H1975; *P* < 0.05 for A549). Scratch wound healing test showed that activated RAC1 could enhance the migration ability of lung cancer cells in the plate (*P* < 0.01 for H1975; *P* < 0.05 for A549). However A549 (*P* < 0.01) and H1975 (*P* < 0.001) recovers wound more slowly in the presence of RAC1 inhibitor (Fig. 6c, d). The phalloidin staining results showed that RAC1 inhibitor could significantly inhibit the expression of polymeric actin in lung cancer (*P* < 0.01), and RAC1 agonist agent could significantly promote the expression of polymeric actin in lung cancer cells (*P* < 0.05, Fig. 6e).

**Fig. 7:**
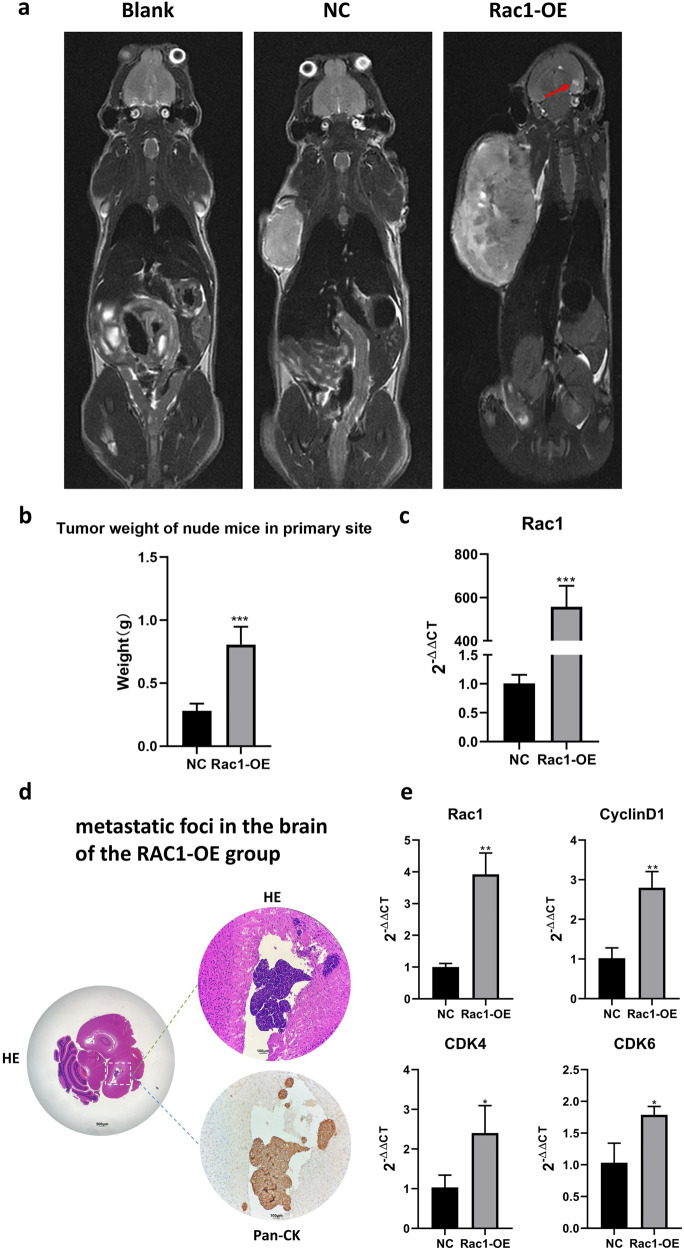
Animal experiment. **a** The MRI T2WI sequences of the Blank and NC groups did not show high signal shadows in the brain parenchyma. The MRI T2WI sequences of the RAC1-OE group showed high signal shadows in the brain parenchyma, indicating that the lung cancer cells metastasized to the brain. **b** The primary focal tumor was significantly heavier in the RAC1-OE group than in the NC group (*P* < 0.001). **c** qPCR results showed that RAC1 overexpression H1975 cells were successfully constructed (*P* < 0.001). **d** HE and Pan-CK staining results demonstrated the presence of metastatic foci in the brain of the RAC1-OE group. **e** RT-PCR on brain tissue showed the expression of RAC1, CDK4, CDK6, and CyclinD1 was significantly increased in the RAC1-OE group. Data are expressed as means ± SD; *P < 0.05, **P < 0.01, ***P < 0.001.

Compared to blank group (no tumor burden) both the NC and RAC1-OE groups showed tumor burden in MRI results, and the primary focal tumor was significantly heavier in the RAC1-OE group than the NC group in weight (*P* < 0.001, Fig. 7a, b). qPCR results showed that RAC1 overexpression H1975 cells were successfully constructed (Fig. 7c). Two of five RAC1-OE mice showed high signal shadows in the brain parenchyma, indicating that the lung cancer cells metastasized to the brain using the MRI T2WI sequences. However the MRI T2WI sequences of high signal shadows in the brain parenchyma is negative either in the Blank or NC groups. It is marginally statistically different between RAC1-OE and NC group (*P* = 0.057, Table 2). We carefully studied different tomographies in the RAC1-OE mice and found metastases loci only occurred in the brain, and no metastases were observed in other organs (especially liver, bone or adrenal gland). There are several metastatic loci in the brain HE sections among which the largest was in the right parietal lobe and lateral ventricle, which is in concordance with the MRI imaging result (Fig. 7d). Next, we performed RT-PCR on brain tissue, and consistent with the results of Fig. 5g, the expression of RAC1, CDK4, CDK6, and CyclinD1 was significantly increased in the RAC1-OE group (Fig. 7e).

**Table 2:** Brain metastasis rate of tumor-bearing nude mice.

	Blank group	NC group	RAC1-OE group	*P* value (Chi-square^a^)
Mice with brain metastases	0	0	2	–
Mice without brain metastases	3	5	3	–
Brain metastases (%)	0	0	40	0.057

^a^Comparison between the NC and RAC1-OE groups.

## Discussion

In this study, we performed a comprehensive study over 8 LUAD patient with brain metastasis using SMART-Seq2 scRNA-seq approach, focusing on the significantly increased expression of RAC1 in the metastatic cells. Sequencing analysis elucidated several genes continuously elevated from primary to CTC to metastasis, and visualized the expression levels of metastasis biomarkers in different subpopulations. The molecular network changes and pathways involved in tumor metastasis were further explored. Pathological and cytological studies confirmed that RAC1 is a key gene promoting brain metastasis of LUAD. Our results highlight the potential molecular mechanisms and cellular therapeutic targets of LUAD brain metastasis, which could provide powerful information for better treatment of gene-driven cancer metastases.

Current opinions on the mechanisms of the brain-metastatic cascade include hematogenous dissemination of tumor cells, attachment to microvessel endothelial cells, extravasation into the brain, interaction with the local microenvironment, angiogenesis and intraparenchymal proliferation [16]. Our functional annotation indicated metastatic cell favored genes functioned as regulated cell death and apoptosis as well as promoted macromolecule organization.

Our results shows that CTC is potentially an intermediate stage between primary and tumor, is considered as precursor of tumor metastasis. Studies related to hepatocellular carcinoma have shown that CTCs enter the bloodstream from primary tumors and metastatic lesions and promote tumor metastasis through EMT [17]. We further expanded the dataset and investigated Epithelial markers, Mesenchymal markers and EMT related pathways hallmarks [18–22] using 452 cells (272 cells from metastatic cancer cells and 180 from primary cancer cells) of all 8 patients. Results revealed a higher Mesenchymal markers in primary cancer cells (e.g., NCAM1, LAMB1, SERPINE1, TCF4, VIM, ZEB1, and ZEB2) and a higher Epithelial markers in metastatic cancer cells (e.g., CDH1, CLDN4, ELF3, EPCAM, KRT18, KRT7, and KRT8). These inducers can downregulate E-cadherin and upregulate N-cadherin and vimentin (VIM) through modulating EMT-related signaling pathways, for instance WNT/β-catenin and TGF-β, and EMT transcription factors, such as zinc finger E-box binding homeobox 1 (ZEB1) and ZEB2 [20]. Elevated oncogenic signaling of TCF/β-catenin in intestinal epithelium, important for tumor progression via disruption of cell polarity and adhesion [23]. Occludin (the OCLN gene) encodes an integral membrane protein that is required for cytokine-induced regulation of the tight junctions (TJs) while KRT8 and KRT19 are two oncogenes important in development of human cancers, and KRT5, KRT14, and KRT17 overexpression are related to poor survival across different types of cancers [24]. We found that the activity of these genes in the metastatic foci is very high, while the primary foci are low, which reflects that the primary the cell structure of primary loci has been subjected to a certain degree of integrity disruption. However, at the metastasis site, it is necessary to recruit these genes to establish cell adhesions and remodeling of the cycloskeleton. It is interesting a few EMT Pathway signaling molecules were upregulated in metastatic cells including AKT1, MAPK1, MAPK3, RAS genes (NRAS, KRAS, and HRAS), ROS1, and PTK2 were up-regulated. Many of the genes play critical roles in cell proliferation, invasion and adhesion such as PTK2 a focal adhesion kinase (FAK) [25].

In the cell transitional state analysis, we identified LCN2, RAC1 and SAT1 were gradually enriched in the metastatic cells. LCN2 promotes inflammatory breast cancer tumorigenesis, skin invasion, and mediate aggressive breast cancer metastasis to distant organs [26, 27]. ASMTL-AS1 impedes the malignant progression of lung adenocarcinoma by regulating SAT1 to promote ferroptosis [28]. Through functional annotation we could infer in the metastatic sites cancer cells need to cope with harsh environment than primary site such as cell death and apoptosis, which further stimulated macromolecule organization and synthesis.

RAC1 plays a major regulatory role in the basis of malignant tumor development, including tumor angiogenesis and invasion/metastasis. High expression of RAC1, RHOA, CDC42, and VEGFB were identified in metastatic subgroups, compared to primary tumor and normal lung epithelia clusters. RHO, RAC1, and CDC42 are all members of the RHO GTPase family and are involved in the regulation of cytoskeleton, cell growth, and cell cycle, which may be effective targets against malignant tumor metastasis [29, 30]. RAC1 is involved in invadopodia-mediated ECM degradation and drives locomotion by regulating patellar pseudopodia formation, adhesion plaques, and MMP expression, which is an activity required for cell migration/invasion during cancer metastasis [31]. Research has been suggested that the combination of VEGF/VEGFR-targeted therapy with RAC1 inhibitor can improve the efficacy of anti-metastasis therapy in prostate cancer [32].

Interestingly, through co-network analyses we found that MUC1, MUC20, CEACAM5, and CCL20 were positively correlated to expression of RAC1, whereas TIMP3 and MGP were negatively correlated to RAC1. Both MUC1 and MUC20 are genes encoding transmembrane mucins that have been identified in the lung. They associated with the potential for metastasis and invasion of tumors [33, 34]. MUC1 regulates cytoskeletal recombination and directs cell movement during cell migration through the MUC1-SrC-CRKL-RAC1/CDC42 cascade [35]. TIMP3 and MGP can inhibit tumor growth, invasion, metastasis and angiogenesis [36, 37]. In addition, functional clustering and pathway analysis showed that genes in RAC1-highly expressed cells played important roles in adhesion, ECM and VEGF signaling pathways and are involved in cell cycle regulation. Our results suggest that RAC1 prevents further cancer progression by blocking the transition from G0/G1 phase to S phase.

RAC1 can promotes the invasiveness and migration ability of LUAD cells and similar results were found in other tumor cell lines. Rac1-induced ROS generation affects actin cytoskeleton reorganization, leading to cell migration and invasion of various tumor cells. Mouse models have shown that downregulation of Rac1/ROS downstream WAVE2 expression inhibits migration and invasion of B16 melanoma cells [38]. In our study, brain metastasis occurred in the RAC1-OE group relative to the NC group. However, due to the sample size limitation, this result is only marginally significant (*P* = 0.057), and we will add more samples in future experiments to provide stronger evidence that RAC1 promotes brain metastasis.

In the present study we obtained a more comprehensive picture over lung cancer metastasis in the single-cell level, giving a new perspective to the role of RAC1 in the LUAD brain metastasis, and related pathways to participate in the metastasis process. This study provides solid evidence for further unraveling the mechanisms of action in treatment of the LUAD metastasis.

## Materials (or subjects) and methods

### Study design and participants

This study is based on a comparative sequencing analysis using single-cell transcriptomic data from one LUAD patient and then expanded to 8 patients who either donated primary tumor biopsy, metastasis tumor tissue, para-metastasis tissue, CTCs from blood, or all of the tissues. The patients were not treated previously by radio therapy, chemotherapy or targeted therapy. The pathology sections from of another 6 LUAD patients with paired metastatic tumor tissue and primary tumor tissue were collected for RAC1 IHC staining. Brain samples were collected from Department of Neurosurgery Huashan Hospital during operation, while primary LUAD tissues and blood were taken from Department of Lung Cancer Shanghai Chest Hospital through biopsy. The study was approved by both of the Ethical Committee in Huashan Hospital and Shanghai Chest Hospital. All the patients enrolled in this study agreed and signed informed consent.

### Solid tumor decomposition and single-cell isolation

Primary and metastatic tissues from biopsy and surgical mass were dissected and transferred to a 2 ml tube (Axygen, China) each containing 1 ml pre-warmed M199-media (ThermoFisher Scientific, USA), 2 mg/ml collagenase P, 2.5 mg collagenase D (Roche, USA) and 10U/μl DNase I (Roche, USA) as was adapted from Tirosh et al.’s protocol [39]. Tissues were digested for 60 min at 37 °C and then pipetted up and down every 10 times every 10 min. The tissue suspensions were then filtered with a 70 μm nylon mesh (ThermoFisher Scientific, USA) and centrifuged at 450 × *g* for 5 min. Pellet was resuspended for live cell staining using CFSE incubation for 5 min.

### CTC Isolation

CTCs were captured by leukocyte negative enrichment using magnetic beads and EpCAM positive staining protocol [40–42]. 7.5 ml peripheral blood (with CTCs spike-in) was collected one hour before brain operation and added onto 30 ml of red blood cell lysis buffer, vortexed for 2 min, stand on ice for 5 min and centrifuged at 450 × *g* for 5 min. Nucleated cell pallet was resuspended and incubated with 350 μl of anti-WBC immunomagnetic beads (human CD15 and CD45 Dyanabeads, Invitrogen) for 20 min. Beads were removed by magnetic rack and the remnant was centrifuged at 450 × *g* for 5 min. Pellet was resuspended for staining using CD45 (CD45-Alexa Fluor 594, Biolegend) and EpCAM antibody (CD326-Alexa Fluor 488, Biolegend) in PBS. CTCs and WBCs were identified and isolated under fluorescent microscopy (X71, Olympus, Japan).

### Single-cell whole-transcriptome library preparation and sequencing

Single cells from tissue were manually picked under fluorescent microscopy using mouth pipette. Each of the harvested single cells was transferred into 2 μl of cell lysis buffer (CLB) in 0.2 ml PCR tubes. Libraries of isolated single cells were then prepared as per Smart-seq2 protocol [43] with modifications on reverse transcription and amplification cycles. Briefly, oligo-dT primed reverse transcription was performed with Smartscribe (Takara, Japan) reverse transcriptase and locked TSO oligonucleotide (Exiqon, Denmark) upon single cells. Full-length cDNA amplification was conducted by 20 cycles of PCR amplification with HiFi-HotStart ReadyMix (KAPA Biosystems, USA) and subsequent 0.6× AMPure beads purification (BD, USA). Barcoded libraries were fragmented and tagmented with Nextera XT Library Prep kit (Illumina). Pooled libraries with unique N5-N7 barcodes were sequenced using a Hiseq 2500 sequencer (Illumina) and single-end 50 bp reads flow cell.

### ScRNA-seq data analysis

Raw data were first processed to remove sequencing adapters and low quality reads using Trimmomatic [44]. Reads with an average quality score of less than 20 bp, and sequencing lengths less than 18 bp were discarded after trimming. The remaining high quality reads were aligned to the human genome using HiSat2 tool [45], by using human genome UCSC hg19 as a reference (ftp://genome-ftp.cse.ucsc.edu/goldenPath /hg19/chromosomes/) with a total 22,335 genes. The FeatureCounts software [46] was used for quantitation of expression for each gene, raw counts values of each sample were obtained. In our analysis, a gene was consider to be expressed in a sample should have one more counts in the sample. Reads counts were normalized to transcript per million (TPM) values and then log2 transformed by used “newSCESet” function of “scater” (https://github.com/davismcc/scater) package of R-project (R3.5.2, https://www.r-project.org/). Raw data with counts were processed with *Seurat* 2.3 (http://satijalab.org/) for heatmap and tSNE plots [47]. Expression matrix for all normalized cells were provided. Analyses, such as Principal component analysis (PCA), Pearson correlation, and Student’s *t* test were performed using functions in R as follows: “prcomp,” “cor,” and “t.test” in the “stats” package, and Heatmap in “ComplexHeatmap” package. The “ggplot2” package was used for the visualization of graphs.

### Differentially expressed genes (DEGs)

DEGs were identified by calculating fold-change and *P* value between “treatment” and control groups. We set a twofold cutoff of fold change and FDR adjusted *P* < 0.05 as the criteria for DEG selection. This was done by using the “stat” package of R project. The KEGG pathway analysis and Gene Ontology (GO) analysis was performed using “KEGGprofile” and “Clusterprofiler” in R.

### Gene co-expression networks (Co-network)

Co-network were built according to the normalized gene expression values, genes with an average expression value across samples larger than the median expression value of all the genes were considered for co-expression analysis. We construct the network adjacency between two genes, *i* and *j*, defined as a power of the Pearson correlation between the corresponding gene expression profiles. By computing the correlation coefficient of these genes, we obtained the gene-to-gene co-expression adjacency matrix. We selected the genes with high correlations (0.8 or greater) to draw a co-expression network graph with CytoScape (v3.4) [48]. Molecular validation of key genes in co-expression networks using qPCR.

### Pseudo-time trajectory construction

To investigate the progression trajectory of LUAD cell, we used minimal spanning tree algorithm-based *monocle* (v2) R-package to perform a pseudo-time analysis.

### RAC1 quantitation in HPA database

The images and expression level of RAC1 in LUAD and normal tissues based on immunohistochemical datasets were explored using the HPA database (https://www.proteinatlas.org/).

### Survival analysis of RAC1 expression status

Survival analysis of LUAD patients with high and low expression of RAC1 in The Cancer Genome Atlas (TCGA) were further analyzed by Oncolnc tool (http://www.oncolnc.org/). Kaplan–Meier survival curve was plotted by using R and *survival* package.

### Immunohistochemistry (IHC) staining

Tissue samples from seven patients with brain metastases from lung adenocarcinoma were collected for IHC staining to examine the expression changes of RAC1 (AF4200, Affinity).

### Cell lines and reagent for functional assay

LUAD A549 and H1975 (CinoAsia Co., Ltd.) were employed for cell line validations. Cells were seeded in 6-well culture plate (2 × 10^5^/well) and cultured in cell incubator until the confluency degree at 90%. RAC1 agonist (CN02-A, 0.5 units/ml, Cytoskeleton, USA) and RAC1 inhibitor (1177865-17-6, 50 μM, Topscience, China) were added into the cells and incubated at 37 °C for 48 h.

### Apoptosis analyses

Briefly 5 μl Annexin V-FITC (BD, USA) and 5 μl Propidium Iodide were mixed with 100 μl resuspended cells. The cells were incubated for 20 min at room temperature away from light. PBS buffer was used wash the cells twice and resuspend cells to 500 μl. At least 2 × 10^4^ cells were analyzed by fluorescent flow cytometry (CytoFlex, Beckman, USA).

### Cell cycle analyses

Cells were washed with PBS and incubated with propidium iodide solution (500 μg/ml and 100 μg/ml RNaseA) for 30 min. Cells were further analyzed by flow cytometry. In addition, qPCR and WB were used to verify the effect of RAC1 (AF4200, Affinity, China) on the expression of cell cycle related genes and proteins CDK4(DF6102, Affinity, China), CDK6 (DF6448, Affinity, China), CyclinD1 (AF0931, Affinity, China).

### Phalloidin staining

Morphological changes were observed by phalloidin (G1041-50UL, Servicebio, China) staining human lung adenocarcinoma cells (H1975) treated with RAC1 agonists and inhibitors with reference to existing methods [49].

### Cell counting kit-8 (CCK-8) proliferation assay

CCK-8 was added to each well after cells were activated or interfered for 24 h, 48 h, and 72 h. OD value was measured by microplate reader (uQuant, BioTek, USA) at 450 nm.

### Transwell assay

Cells were seed at upper microholed wells with Matrigel plated. The wells were placed into the lower well containing 500 µl of complete medium. 37 °C, 12 h, cells were gently removed in the upper wells with a cotton swab. The cells in the lower chamber were fixed for 10 min and stained with 1% crystal violet dye in 2% ethanol for 15 min.

### Wound-healing assay

A pipette tip was used to scratch a line on the cell layer when the cells reached at 90% confluency. The picture of each wound was recorded by microscope after 0 and 24 h. Image J software (v1.50i) was used to calculate the mean value of the distance of each wound.

### Construction of RAC1 overexpressing H1975

The sequence of the CDS region of the human Rac1 gene was inserted into the lentiviral plasmid FV115 to synthesize the Rac1 overexpression lentiviral plasmid (GeneralL Biol, China). H1975 cells were inoculated into 6-well plates at 1 × 10^6^ cells/well, and the Rac1 overexpression lentiviral plasmid was transfected into H1975 cells using HilyMAX reagent (Dojindo, Japan) according to the manufacturer’s instructions. The transfected cells were then placed in a 37 °C, 5% CO_2_ cell culture incubator, and stable Rac1 overexpressing H1975 cells were selected and expanded in RPMI1640 complete medium containing 200 μg/ml G418. qPCR verify the efficiency of RAC1 overexpression.

### Nude mice subcutaneous tumor model and magnetic resonance imaging (MRI) observation of brain metastasis

Nude mice random divided into three groups. Blank group: three 6-week-old male Balb/c-nu nude mice (Shanghai SLAC Laboratory Animal Co.,Ltd, Shanghai, China) without any treatment. NC group: five 6-week-old male Balb/c-nu nude mice injected subcutaneously with H1975. Rac-1 OE: five 6-week-old male Balb/c-nu nude mice subcutaneously injected with RAC1 over-expression H1975. 2 × 10^6^ cells were mixed in 100 μl serum-free RPMI-1640 medium and injected subcutaneously into the right axilla of nude mice, which were kept in an SPF class animal house for 6 weeks. Mice were examined using a Magnetic Resonance Imaging (uMR790, 3.0 T) (United-imaging, China). T2-weighted MRI images were used for data analyses. All procedures involved in mice complied with the protocol of Animal Ethics Committee of Huashan Hospital.

### HE and Pan-CK staining and qPCR of brain tissue in model mice

The brain tissue of model mice were obtained every 5 μm per step until HE staining revealed potential metastasis sites. The sections stained with hematoxylin solution for 5 min and put in 1% acid ethanol (1% HCl in 70% ethanol) and then rinsed in distilled water. Then stained with eosin solution for 2 min and followed by dehydration with graded alcohol and clearing in xylene. Pan-CK (Bioss, bs-1712R) staining was further implemented for the confirmation of cancer cells in the brain. In addition, RT-PCR were used to verify the expression level of RAC1, CDK4, CDK6, CyclinD1.

### Statistical methods

Data were expressed as means ± standard deviation (SD) and analyzed using R or base statistical package. Unpaired Student’s *t* tests were used to compare the means of two groups. Statistics were performed using Prism 8 (GraphPad, San Diego, USA) and significance is indicated in the figures (*P < 0.05, **P < 0.01, ***P < 0.001). A *P* value <0.05 was considered statistically significant. Pearson correlation coefficient was used for correlation analysis.

## Supplementary information

supplementary table 1

Supplementary figures

aj-checklist

original western blot-ACTIN

original western blot-CDK4

original western blot-CDK6

original western blot-Cyclin D1

original western blot-Rac1

## Data Availability

The datasets generated and/or analyzed during the current study are available in the [Gene Expression Omnibus] repository https://www.ncbi.nlm.nih.gov/geo/query/acc.cgi?acc=GSE198291.
