# Supplementary material for: Identification of RAC1 in promoting brain metastasis of lung adenocarcinoma using single-cell transcriptome sequencing
Source: Cell Death Dis. 2023 May 18;14(5):330. doi: 10.1038/s41419-023-05823-y (PMC10195834; doi:10.1038/s41419-023-05823-y)
Supplement: Supplementary file 1 — supplementary table 1 [file 41419_2023_5823_MOESM1_ESM.docx]

Table S1. Metastasis-to-CTC-to-primary continuously upregulated genes

|  | meanLogTPM_PTT | meanCTC | meanMTT | pvaluecp | pvaluemc | CminusP | MminusC | MminusP |
| --- | --- | --- | --- | --- | --- | --- | --- | --- |
| LCN2 | 4.074583842 | 7.207344575 | 7.272221211 | 7.95E-23 | 9.10E-32 | 3.132760734 | 0.064876636 | 3.197637369 |
| SAT1 | 5.321456582 | 7.832285471 | 10.23747348 | 5.04E-22 | 3.99E-41 | 2.510828889 | 2.405188006 | 4.916016895 |
| RAC1 | 4.893182415 | 6.436048644 | 8.483342955 | 8.09E-24 | 6.19E-35 | 1.542866228 | 2.047294312 | 3.59016054 |
| IFITM3 | 5.613327561 | 6.55525231 | 9.837702039 | 1.67E-24 | 1.20E-36 | 0.941924749 | 3.282449729 | 4.224374478 |
| VAMP8 | 4.259014321 | 4.999632082 | 7.612416686 | 7.74E-27 | 9.21E-32 | 0.740617761 | 2.612784604 | 3.353402365 |
| RAB13 | 4.151497652 | 4.890001604 | 7.781604477 | 2.44E-26 | 9.77E-35 | 0.738503952 | 2.891602874 | 3.630106826 |
| OFD1 | 4.388119311 | 5.124330209 | 7.460001947 | 2.08E-25 | 1.05E-28 | 0.736210898 | 2.335671738 | 3.071882635 |
| TMSB10 | 7.919133788 | 8.526388816 | 11.51354672 | 4.38E-29 | 3.75E-51 | 0.607255028 | 2.987157905 | 3.594412933 |
| CRIP1 | 4.071658631 | 4.609960574 | 6.696897285 | 3.70E-27 | 5.57E-30 | 0.538301943 | 2.086936711 | 2.625238654 |
| DCP1A | 3.859055129 | 4.339851255 | 4.352240012 | 1.90E-31 | 1.25E-33 | 0.480796126 | 0.012388757 | 0.493184883 |
| NFKBIA | 5.073400148 | 5.540880327 | 9.713013789 | 1.20E-22 | 8.98E-32 | 0.46748018 | 4.172133462 | 4.639613642 |
| S100A4 | 4.483500937 | 4.898957128 | 7.632333059 | 1.85E-24 | 1.64E-28 | 0.415456191 | 2.733375931 | 3.148832122 |
| MIF | 7.567735372 | 7.943291733 | 10.7112598 | 1.90E-24 | 1.07E-40 | 0.375556361 | 2.767968066 | 3.143524427 |
| GDF15 | 4.150013614 | 4.500179767 | 7.082641605 | 1.36E-25 | 1.92E-27 | 0.350166153 | 2.582461838 | 2.932627991 |
| S100A10 | 5.046683067 | 5.385987447 | 8.204743352 | 1.64E-23 | 6.27E-32 | 0.33930438 | 2.818755905 | 3.158060285 |
| MUC1 | 4.005188561 | 4.312620992 | 7.650462413 | 4.75E-28 | 1.14E-27 | 0.307432431 | 3.33784142 | 3.645273851 |
| CCL3 | 3.923299158 | 4.203363656 | 4.318141772 | 2.93E-29 | 1.15E-32 | 0.280064498 | 0.114778116 | 0.394842614 |
| CSRP1 | 3.88254824 | 4.132709363 | 4.913624644 | 7.94E-33 | 9.50E-34 | 0.250161123 | 0.780915281 | 1.031076404 |
| IGFBP7 | 4.08222644 | 4.327328529 | 5.006852277 | 2.85E-26 | 6.59E-30 | 0.245102089 | 0.679523748 | 0.924625837 |
| ARPC5L | 4.095717241 | 4.307292898 | 4.83653112 | 2.91E-27 | 2.04E-30 | 0.211575658 | 0.529238221 | 0.740813879 |
| SOX4 | 5.654066283 | 5.862517015 | 8.627740263 | 8.58E-23 | 1.45E-32 | 0.208450732 | 2.765223248 | 2.973673979 |
| COL4A2 | 4.028183821 | 4.23042081 | 4.481626734 | 2.34E-28 | 7.28E-31 | 0.202236988 | 0.251205925 | 0.453442913 |
| SERPINF1 | 3.826669773 | 3.986483936 | 4.123950129 | 3.62E-35 | 1.45E-35 | 0.159814163 | 0.137466193 | 0.297280356 |
| FSTL1 | 4.096976654 | 4.244682896 | 4.622386411 | 8.80E-28 | 2.41E-31 | 0.147706242 | 0.377703515 | 0.525409757 |
| IKBKG | 3.686572417 | 3.825555942 | 3.906472025 | 2.38E-44 | 4.71E-51 | 0.138983525 | 0.080916083 | 0.219899608 |
| TPM2 | 3.587907672 | 3.701875503 | 3.86604271 | 1.54E-69 | 1.88E-42 | 0.113967831 | 0.164167207 | 0.278135038 |
| RSPO1 | 5.041808839 | 5.126554775 | 9.7304915 | 1.65E-24 | 1.93E-38 | 0.084745936 | 4.603936726 | 4.688682661 |
| S100A11 | 5.58192857 | 5.639607918 | 10.01591122 | 4.94E-24 | 2.87E-37 | 0.057679348 | 4.376303303 | 4.43398265 |
| SERPINH1 | 4.211592982 | 4.252022714 | 4.404046233 | 1.67E-27 | 1.86E-33 | 0.040429732 | 0.152023519 | 0.192453251 |
| ST14 | 3.78970856 | 3.812242463 | 6.444683135 | 7.25E-36 | 5.27E-28 | 0.022533903 | 2.632440672 | 2.654974575 |
