# Supplementary material for: Identification of RAC1 in promoting brain metastasis of lung adenocarcinoma using single-cell transcriptome sequencing
Source: Cell Death Dis. 2023 May 18;14(5):330. doi: 10.1038/s41419-023-05823-y (PMC10195834; doi:10.1038/s41419-023-05823-y)
Supplement: Supplementary file 2 — Supplementary figures [file 41419_2023_5823_MOESM2_ESM.docx]

**Supplementary figures**


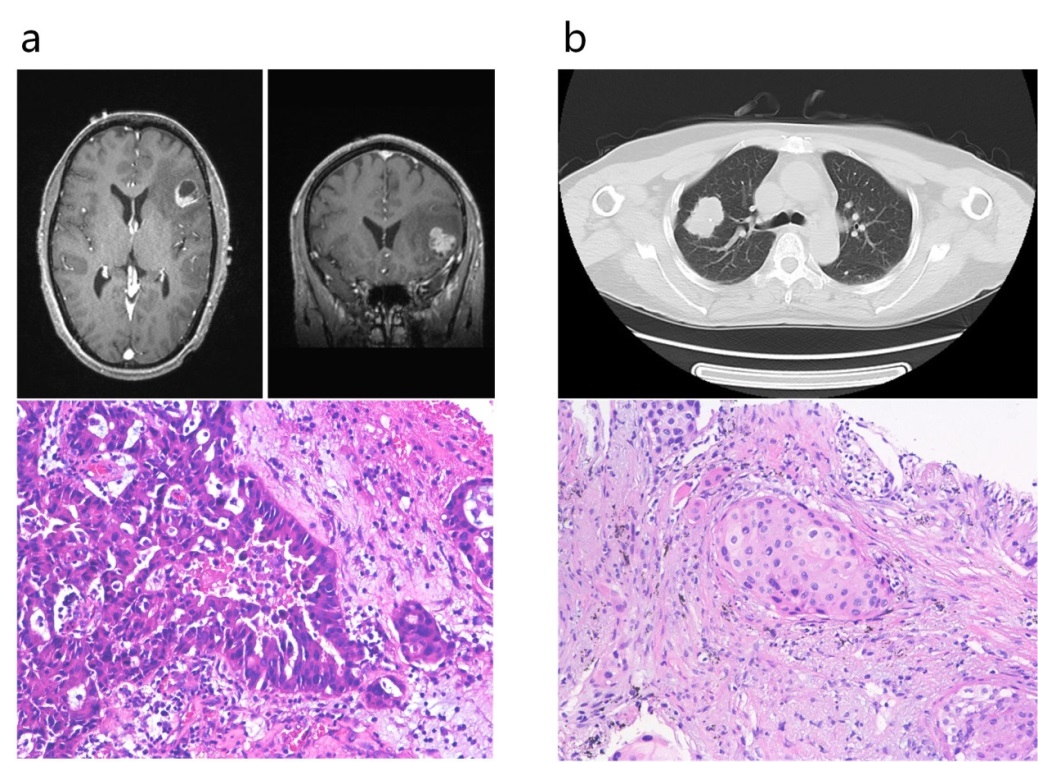


Figure S1. Crainial image and histology sections showed mid-low differentiated morphology of cancer cells of metastatic tumor in the brain (a) and primary cancer in the lung (b).


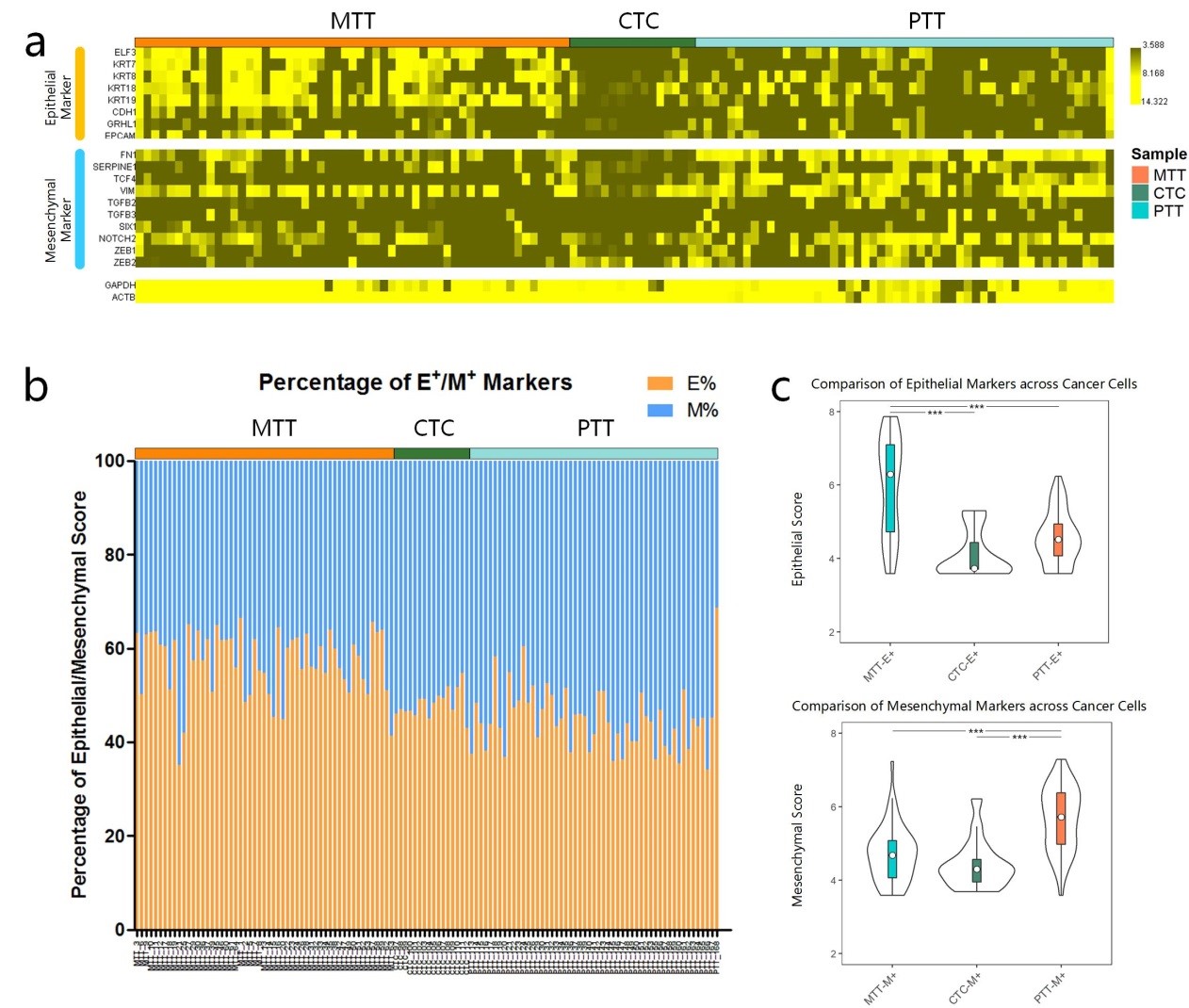


Figure S2. EMT analysis of cancer cells. (a) Heatmap of Epithelial and Mesenchymal markers across all cancer cells. (b) E/M marker percentage of each cell was plotted indicating that a higher mesenchymal state was shown in primary tumor tissue but (c) MTT showed propensity to epithelial state while PTT are more mesenchymal.


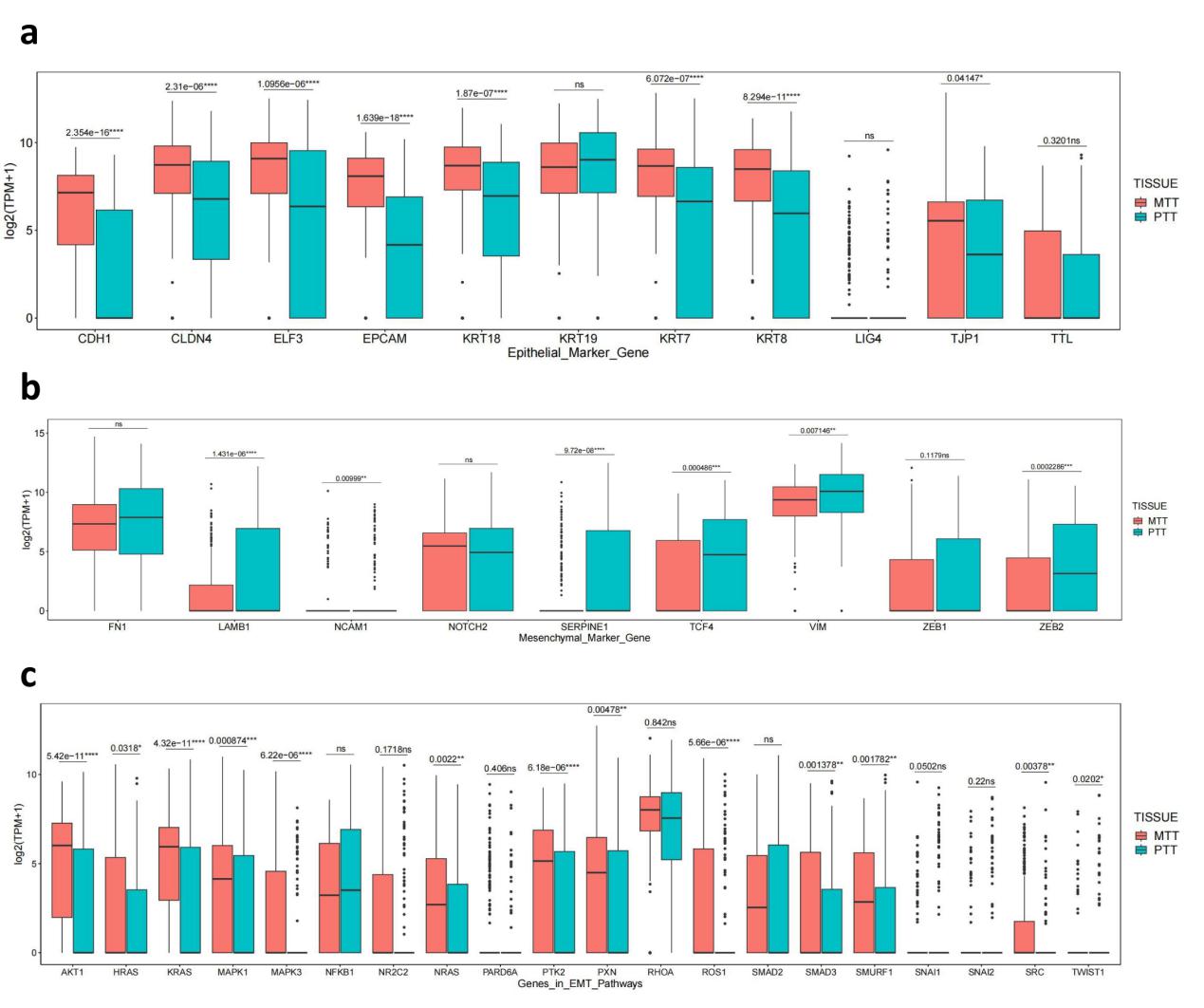
Figure S3. EMT signature analyses in single cancer cells from 8 patients. We further expanded the dataset and investigated Epithelial markers, Mesenchymal markers and EMT related pathways hallmarks using 452 cells of all 8 patients. a) Higher Epithelial markers in metastatic cancer cells (e.g. CDH1, CLDN4, ELF3, EPCAM, KRT18, KRT7, and KRT8) and b) Higher Mesenchymal markers in primary cancer cells (e.g. NCAM1, LAMB1, SERPINE1, TCF4, VIM, ZEB1, and ZEB2) were observed. c) The EMT Pathway signaling molecules were upregulated in metastatic cells including AKT1, MAPK1, MAPK3, RAS genes (NRAS, KRAS, and HRAS), ROS1, and PTK2.


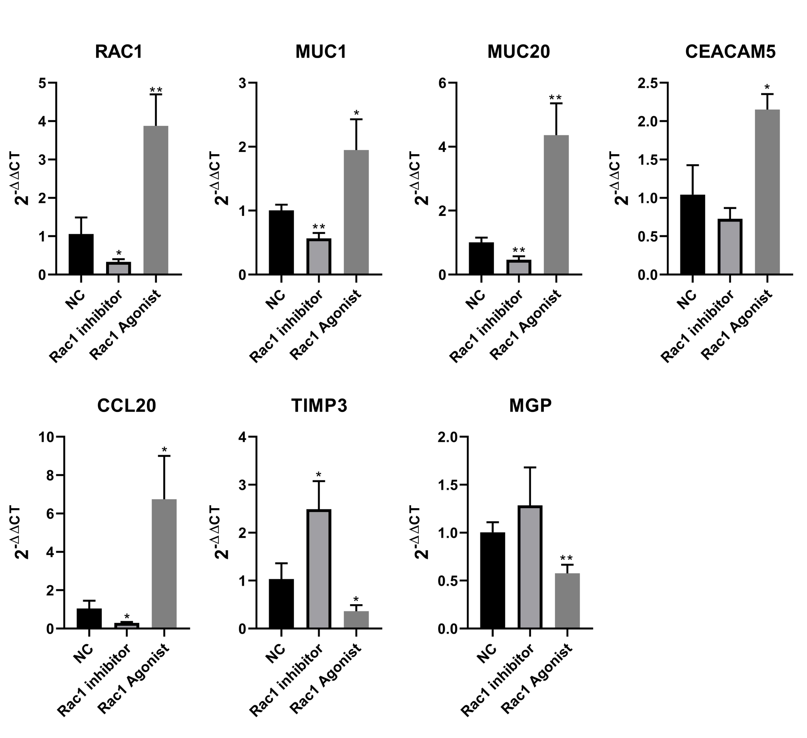


Figure S4. qPCR to verify that RAC1 expression was positively correlated with MUC1, MUC20, CEACAM5, and CCL20, and negatively correlated with TIMP3 and MGP.


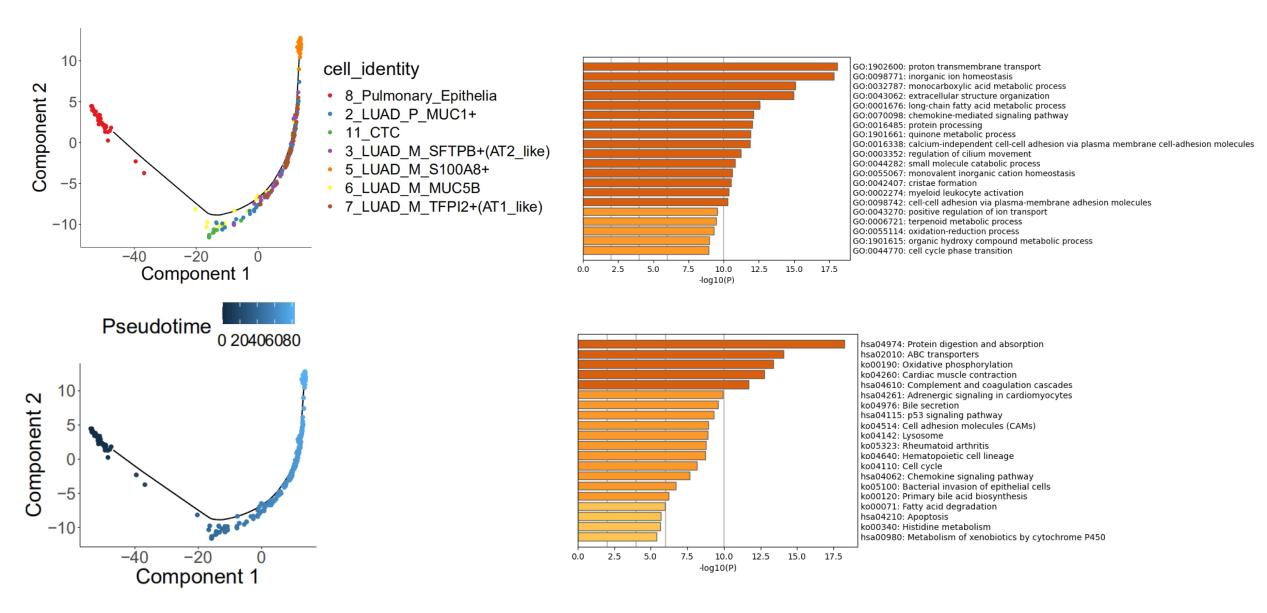


Figure S5. To demonstrate the evolution of LUAD, we plotted a graph based on Monocle 2. The solid black line represented the main route of the minimal spanning tree constructed, which exhibited the backbone and order of the LUAD development along a pseudo-temporal continuum. The trajectory clearly showed the tree starts from 8_Pulmonary_Epithelis, goes through subgroups 6, 11, 3, 2, 7 and ends with 5_LUAD_M_S100A8. Based on the pseudo-temporal continuum profile, we identified representative GOBP and KEGG concentrating terms based on regulated kernel evolutionary enriched genes functional annotations.
