# Supplementary figures and images for: Identification of RAC1 in promoting brain metastasis of lung adenocarcinoma using single-cell transcriptome sequencing
Source: Cell Death Dis. 2023 May 18;14(5):330. doi: 10.1038/s41419-023-05823-y (PMC10195834; doi:10.1038/s41419-023-05823-y)

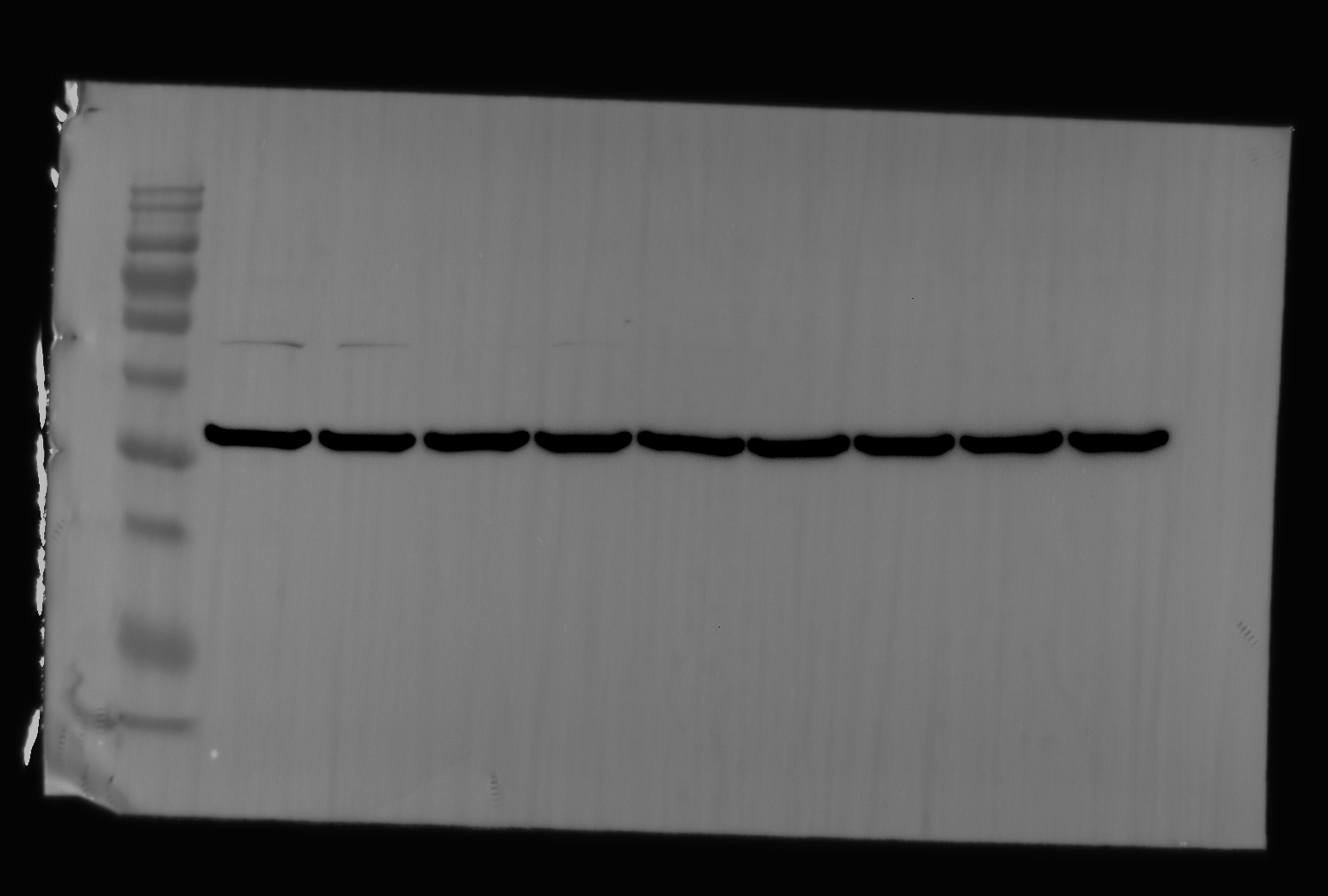

Supplement: Supplementary file 4 — original western blot-ACTIN [file 41419_2023_5823_MOESM4_ESM.tif]

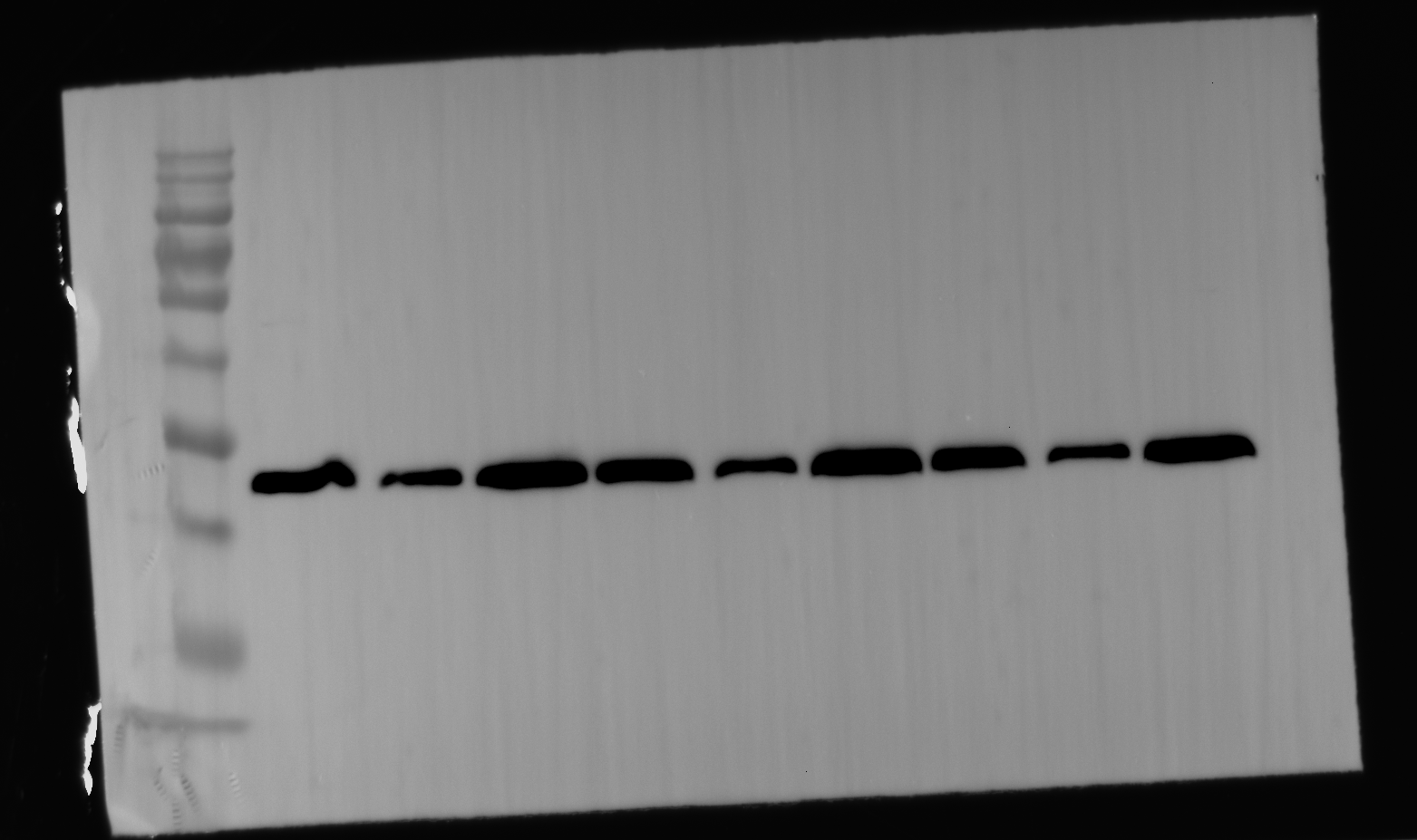

Supplement: Supplementary file 5 — original western blot-CDK4 [file 41419_2023_5823_MOESM5_ESM.tif]

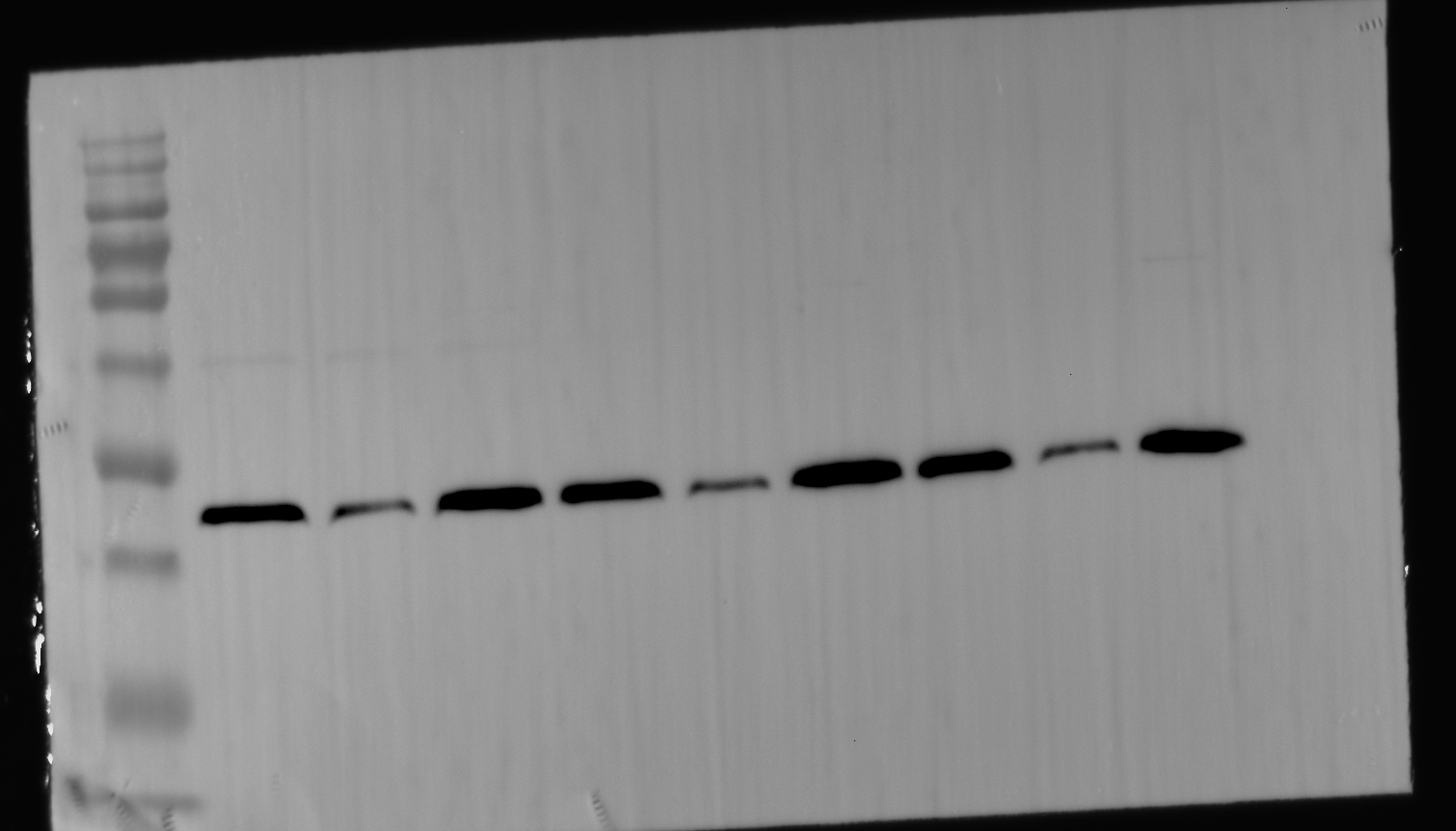

Supplement: Supplementary file 6 — original western blot-CDK6 [file 41419_2023_5823_MOESM6_ESM.tif]

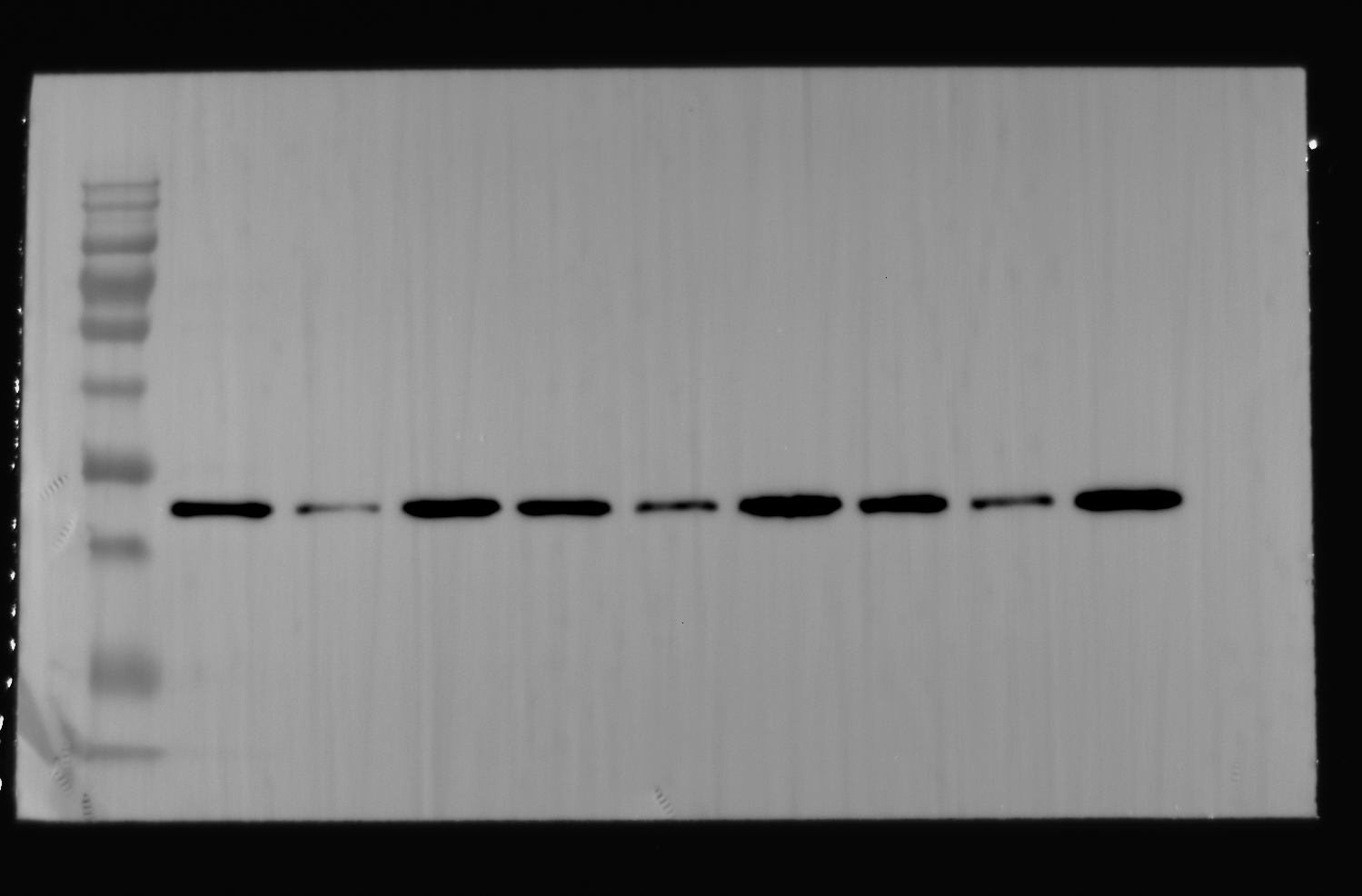

Supplement: Supplementary file 7 — original western blot-Cyclin D1 [file 41419_2023_5823_MOESM7_ESM.tif]

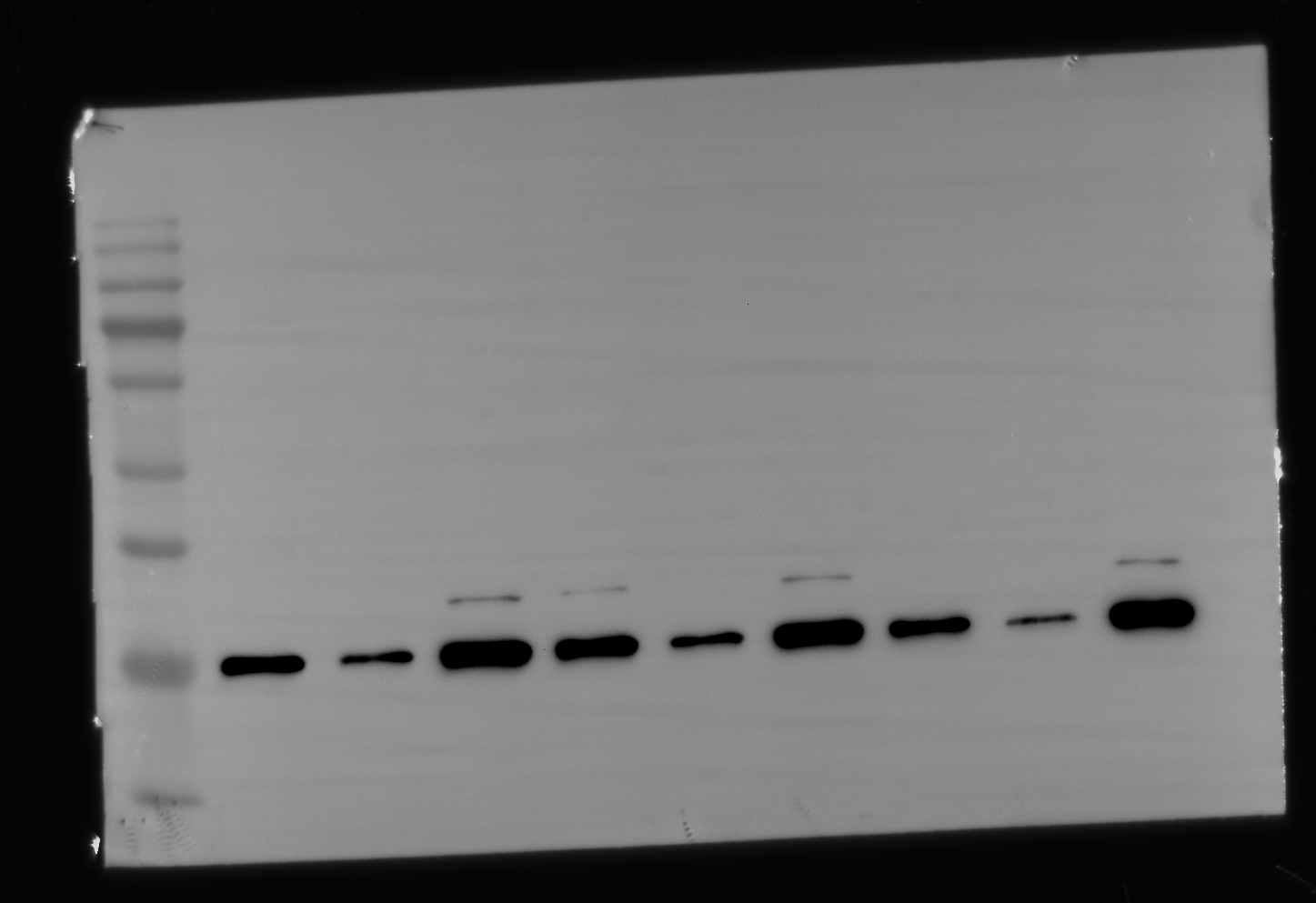

Supplement: Supplementary file 8 — original western blot-Rac1 [file 41419_2023_5823_MOESM8_ESM.tif]
